# Direct Observation of Interfacial Dzyaloshinskii-Moriya Interaction from Asymmetric Spin-wave Propagation in W/CoFeB/SiO_2_ Heterostructures Down to Sub-nanometer CoFeB Thickness

**DOI:** 10.1038/srep32592

**Published:** 2016-09-02

**Authors:** Avinash Kumar Chaurasiya, Chandrima Banerjee, Santanu Pan, Sourav Sahoo, Samiran Choudhury, Jaivardhan Sinha, Anjan Barman

**Affiliations:** 1 Department of Condensed Matter Physics and Material Sciences, S. N. Bose National Centre for Basic Sciences, Block JD, Sec. III, Salt Lake, Kolkata 700106, India

## Abstract

Interfacial Dzyaloshinskii-Moriya interaction (IDMI) is important for its roles in stabilizing the skyrmionic lattice as well as soliton-like domain wall motion leading towards new generation spintronic devices. However, achievement and detection of IDMI is often hindered by various spurious effects. Here, we demonstrate the occurrence of IDMI originating primarily from W/CoFeB interface in technologically important W/CoFeB/SiO_2_ heterostructures using Brillouin light scattering technique. Due to the presence of IDMI, we observe asymmetry in the peak frequency and linewidth of the spin-wave spectra in the Damon-Eshbach (DE) geometry at finite *k* wave-vectors. The DMI constant is found to scale as the inverse of CoFeB thickness, over the whole studied thickness range, confirming the presence of IDMI in our system without any extrinsic effects. Importantly, the W/CoFeB interface shows no degradation down to sub-nanometer CoFeB thickness, which would be useful for devices that aim to use pronounced interface effects.

The Dzyaloshinskii-Moriya interaction (DMI)^1^,^2^ has drawn significant interest due to its fundamental nature and application potential in next generation memory devices. The DMI can be classified into two classes, namely, bulk and interfacial, depending on the type of inversion symmetry breaking^3^,^4^. Bulk DMI is determined by the detailed symmetry of the lattice structure and it has been studied mostly for B20 structures such as MnSi, etc^3^,^5^. Due to the complexity of the nature of symmetry at the interface, it is quite non-trivial to get insight into interfacial DMI.

In last few years remarkable progress on fundamental and technological front in the field of spintronics has been made by utilizing ferromagnetic thin film with structure inversion asymmetry. For applications in magnetic memory devices, emerging device concepts based on domain wall motion^6^,^7^,^8^ and skyrmionic lattice^4^ have been proposed, which aim to use heavy metal (HM)/ultrathin ferromagnet (FM)/oxide heterostructures. In such heterostructures, due to the strong spin-orbit interaction in HM layer, perpendicular magnetic anisotropy^9^,^10^, Rashba effect^11^,^12^, spin Hall effect^13^,^14^, and interfacial Dzyaloshinskii-Moriya interaction (IDMI)^15^ are manifested. Strong IDMI is the key to stabilize the skyrmionic lattice which has significant technological application potential in ultra-dense information storage. Furthermore, IDMI may lead to soliton like domain wall (DW) motion, which can extend Walker breakdown field, stabilizes chiral magnetic order and increases DW velocities even in the precessional regime^16^.

The direct precise quantitative estimation of IDMI constant and its scaling behavior with ferromagnetic layer thickness variation are crucial for understanding the origin of IDMI in HM/FM/oxide heterostructures. Recent studies have applied Brillouin light scattering (BLS)^17^,^18^ technique for such investigations^19^,^20^,^21^. In this technique, the wave-vector (*k*) of the spin-wave is uniquely defined by the wavelength and incident/scattering angle of the laser beam. The advantage of using BLS is that it can detect propagating spin-wave excitation simultaneously at +*k* and −*k* wave-vectors (Stokes and anti-Stokes process)^18^,^22^. The imprint of IDMI in BLS technique is manifested as an asymmetry in the spin-wave dispersion relation for nonreciprocal propagation of Damon-Eshbach (DE) spin-waves^19^,^23^. In FM thin films with broken inversion symmetry, different surface anisotropy on both sides of film results in slight change in spin-wave frequency upon reversal of propagation direction^24^. Recent theoretical calculations have shown that the difference in frequency due to asymmetric surface pinning is an order of magnitude smaller than that resulting due to IDMI^25^. Furthermore, the symmetric conventional exchange interaction (known as Heisenberg exchange interaction) produces quadratic dispersion, an even function of the wave number, while the antisymmetric DM exchange is characterized by an odd linear functional dependence^26^,^27^,^28^. The experiments manifesting these effects have been mostly confined to the thin film stacks that essentially contain Pt next to the ferromagnetic layer^20^,^23^,^29^,^30^. As the Pt/FM layer interface usually gives rise to significant interface anisotropy as well as interfacial DMI, isolation of both the contributions to the asymmetric spin-wave dispersion in such system is nontrivial. Furthermore, a recent study investigated the ferromagnetic layer thickness dependence of IDMI in Pt/CoFeB/AlOx and found a decrease in IDMI constant below CoFeB thickness of 1.6 nm mainly due to degradation of interface^23^. For application of effects originating from interface, it is essential to reduce the thickness of the CoFeB layer: thinner magnetic layer will likely have more pronounced interface effects. Apart from the ferromagnetic layer, underlayer plays a key role in improved interfacial properties in HM/FM/oxide stack. Hence, search for alternative thin film structures with different HM adjacent layer with FM layer with large IDMI originating primarily from interface is the need of the moment in spintronics research. An important thin film heterosturcture CoFeB/SiO_2_ with W underlayer, due to large spin Hall angle of W^31^, has significant application potential in recently discovered three terminal devices^13^. The advantage of the use of W instead of Pt in magnetic recording industry will be its cost effectiveness. Moreover, the choice of W helps to avoid the complication of induced magnetic moment in Pt which makes it difficult to identify the origin of IDMI.

Here, we report the presence of pure IDMI in W/CoFeB/SiO_2_ heterostructure using BLS spectroscopy for varying thickness values of the CoFeB layer down to 0.85 nm. We study the influence of the IDMI on spin-wave properties, in particular, by investigating the asymmetry in the spin-wave dispersion relation for DE mode with respect to counter propagating directions. Using analytical expressions for asymmetric dispersion of spin-wave, the experimental data are modeled to extract the IDMI constant. Detailed analysis of linewidth reveals that the lifetime of the magnons is also asymmetric with respect to propagation along +*k* and −*k* directions. Most significantly, we find that the IDMI constant varies inversely with the CoFeB thickness down to CoFeB thickness of 0.85 nm, indicating the presence of an almost pure IDMI and absence of other spurious effects contributing to the asymmetric propagation of spin-waves.

## Results

### Magnetic characteristics of the thin films

**Figure 1:**
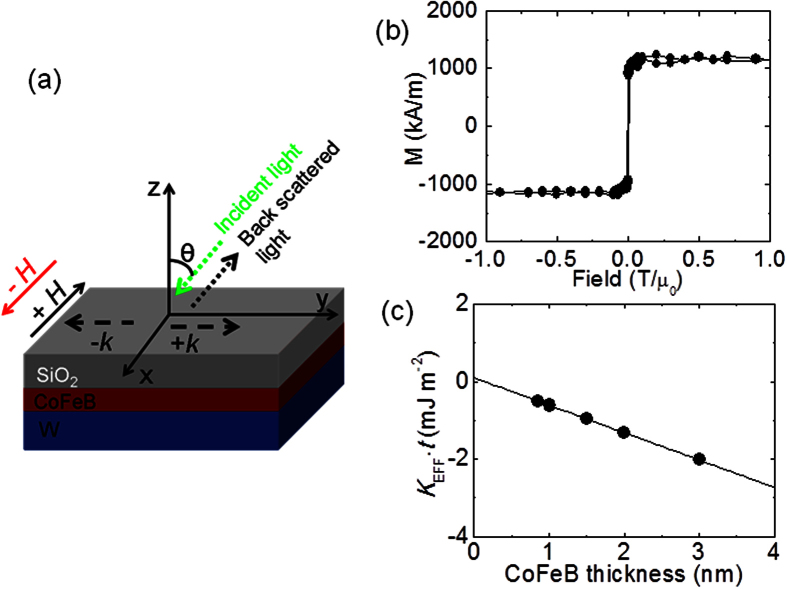
(**a**) Schematic of the film stack along with the BLS measurement geometry. (**b**) Magnetization hysteresis loop for film stack 2 W/1 CoFeB/2 SiO_2_ with magnetic field applied within the film plane. (**c**) The plot of *K*_*EFF*_*.t* as a function of CoFeB thickness along with the linear fit to estimate the interface anisotropy.

Magnetic heterostructures consisting of Substrate/2 W/*t* Co_20_Fe_60_B_20_/2 SiO_2_ (digits represent thickness in nm, subsequently in the manuscript digit is mentioned to indicate thickness) are studied (see methods for details). The BLS experiment was performed in DE^18^,^32^ geometry under the application of an in-plane magnetic field up to ±0.22 T/μ_0_. For the in-plane applied field the magnetization is saturated in the plane of the film. The schematic of the general film stack and BLS experimental geometry is shown in Fig. 1(a). The coordinate convention, the incident light, spin-wave propagation, and applied field directions are indicated. Using Lorentzian function we fit the Stokes and anti-Stokes peaks in the BLS spectra to extract the peak frequency and linewidth i.e., full width at half maximum. Figure 1(b) shows the magnetization hysteresis loop measured by Vibrating Sample Magnetometer (VSM) at room temperature for the film stack Sub/2 W/1 CoFeB/2 SiO_2_ for magnetic field applied within the sample plane thus indicating in-plane easy-axis. The estimated saturation magnetization from the hysteresis loop is 1100 kA/m. All the films have in-plane magnetization as indicated by VSM measurement. We estimate the effective anisotropy (*K*_*EFF*_) of the film by calculating the areal difference between the out of plane and in-plane magnetization hysteresis loop. In Fig. 1(c) we show the plot of *K*_*EFF*_.*t* vs. *t* and use a linear fit to obtain the interface anisotropy (as determined by the intercept on the y-axis). A nearly zero intercept on the y-axis indicates that interface anisotropy is almost negligible in these film stacks.

**Figure 2:**
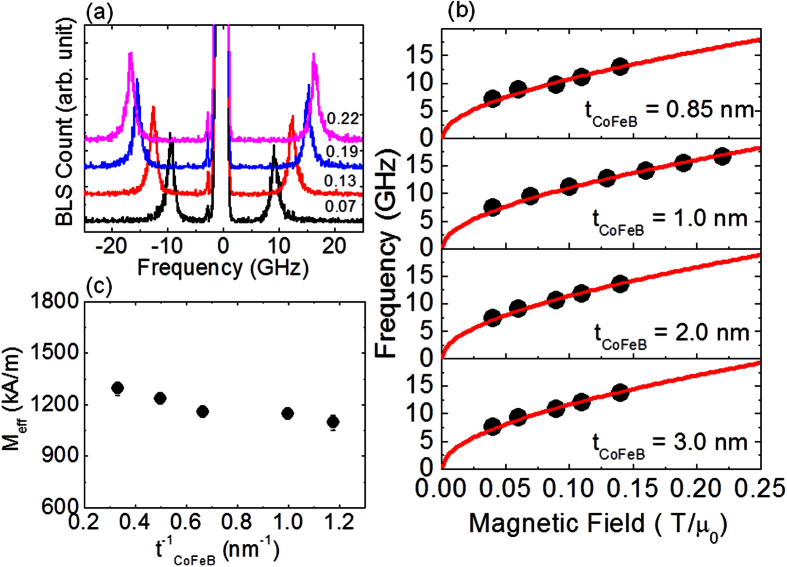
(**a**) Representative BLS spectra measured for the 2 W/1 CoFeB/2 SiO_2_ film stack for various in-plane applied magnetic fields at *k* = 0. Digits mentioned above each spectrum correspond to the magnetic field values in T/μ_0_. (**b**) Plot of frequency versus magnetic field for 2 W/*t* CoFeB/2 SiO_2_ film stack at various *t*. The value of *t* is mentioned in each panel. Symbols represent the experimentally measured data point whereas solid curve is the fit using standard Kittel equation. (**c**) Variation of *M*_*eff*_ as a function of inverse of CoFeB thickness.

Shown in Fig. 2(a) are the typical BLS spectra measured for zero wave-vector (*k* = 0, corresponding to uniform precession mode) at various in-plane applied magnetic fields. The field values are mentioned above each spectra. We use Kittel equation (Eq. (1)) to fit the increase in frequency with the increase in field for different thicknesses of CoFeB (c.f., Fig. 2(b), CoFeB thickness value is mentioned in each plot).


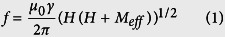


Here, *γ* = *gμ*_*B*_*/h*, *g* is the Lande *g* factor, *H* is the applied bias magnetic field and *M*_*eff*_ is the effective magnetization. We use *μ*_*0*_ = 4π × 10^−7^ N/m^2^ the vacuum permeability and determine g and *M*_*eff*_ as fitting parameter. The best fit to the data yields *g* = 2.00 ± 0.05 for all the thicknesses of CoFeB measured here. Similar measurements of frequency vs. bias magnetic field at *k* = 0 were performed for all other samples and the modeling yields the *M*_*eff*_ parameters as shown in Fig. 2(c) for various thickness of CoFeB. As expected we find that the *M*_*eff*_ shows small variation as the CoFeB thickness is varied thus indicating negligible out of plane anisotropy.

It is important to mention here that observation of asymmetry in the magnon linewidth of the spin-wave spectra is quite non-trivial. In order to observe such asymmetry a major pre-requisite is to select a sample which has reasonably small linewidth. Thus, before measuring the detailed dispersion, the magnetic homogeneity of Sub/d W/1 Co_20_Fe_60_B_20_/2 SiO_2_ is investigated as a function of *d* (data not shown here) by analyzing the linewidth of corresponding BLS spectra at *k* = 0 wave-vector. From this investigation we find that *d* = 2 nm is the optimum W thickness where the linewidth (magnetic inhomogeneity) is reasonably small as well as the frequency asymmetry is large. Thus, for further investigation of asymmetry in frequency vs wave-vector dispersion and asymmetry in the magnon linewidth, we vary CoFeB thickness from 0.85 nm to 3.0 nm by fixing the W underlayer thickness to 2 nm.

### Asymmetry in frequency versus wave-vector due to IDMI

**Figure 3:**
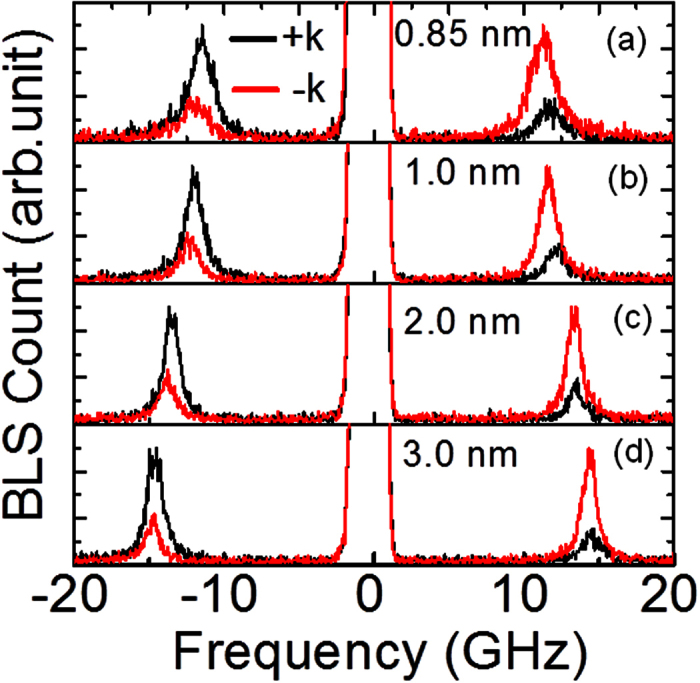
(**a–d**) BLS spectra measured at wave-vector *k* = 2.04 × 10^7^ rad/m for the 2 W/*t* CoFeB/2 SiO_2_ film for two counter propagating directions. The spectrum corresponding to particular thickness of CoFeB is indicated by mentioning thickness value in each panel.

In order to investigate the presence of IDMI in these film stacks, we measured the frequency (*f*) vs. wave-vector (*k*) dispersion by switching the direction of the bias field at a fixed field strength of 0.1 T/μ_0_. The wave-vector (*k*) is varied by changing the angle of incidence of light *w.r.t.* the film plane. Figures 3(a–d) show the typical BLS spectra at a fixed wave-vector *k* = 2.04 × 10^7^ rad/m for various thicknesses of CoFeB in 2 W/*t* CoFeB/2 SiO_2_ stack. From these figures it can be seen that for *t* = 3.0 nm, the peak frequencies for Stokes and anti-Stokes components are nearly the same when the applied field direction is interchanged from +x (black line) to −x (red line). The difference between the frequencies due to field reversal (Δ*f*) for both the Stokes and anti-Stokes lines increases continuously with the reduction in CoFeB thickness as can be noticed in Fig. 3. The value of *Δf* reaches a maximum of 0.5 GHz for *t* = 0.85 nm. Such a large difference in Δ*f* is unlikely to arise from the asymmetric surface pinning of the CoFeB layer in these heterostructures. Moreover, no frequency asymmetry is found in the test sample (1 CoFeB/2 SiO_2_ stack, see methods) which does not have a W underlayer (data not shown). Note that asymmetry in the peak intensity is consistently present at higher *k* values for each film stack with asymmetry more pronounced for films with W underlayer in comparison to that without W.

**Figure 4:**
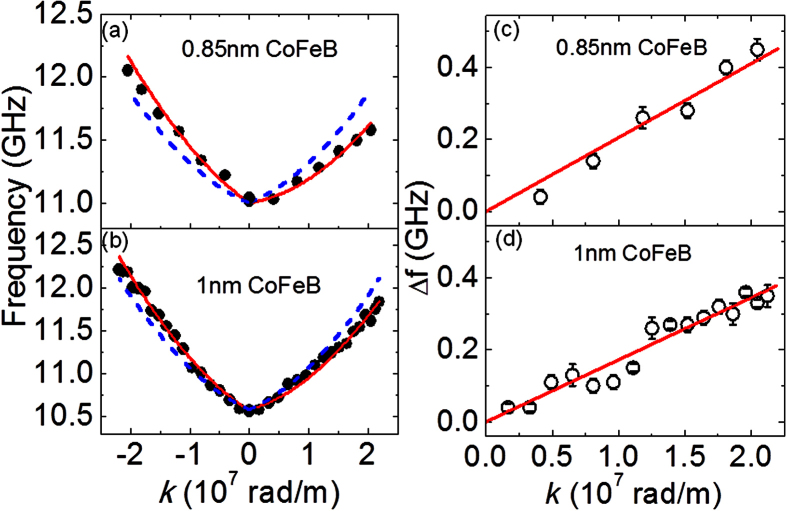
Frequency vs. wave-vector dispersion curves for (**a**) 2 W/0.85 CoFeB/2 SiO_2_ and (**b**) 2 W/1 CoFeB/2 SiO_2_ films. Symbols represent the experimental data points; red solid curves show the fit to the data points using Eq. 2. The blue dashed curves show the dispersion curves in the absence of IDMI. Plot of Δ*f* vs. *k* for (**c**) 2 W/0.85 CoFeB/2 SiO_2_ and (**d**) 2 W/1 CoFeB/2 SiO_2_ films. Here, solid lines represent the linear fit to the experimental data using Eq. 3 to estimate the DMI constant. The error bar in ∆*f* is shown by considering the error from the fitting of the spin-wave spectra.

The full *f* vs. *k* dispersion for two different thickness values of CoFeB (*t* = 0.85 nm and 1.0 nm) are shown in Fig. 4(a,b). The experimentally obtained *f* values are shown using symbols in the figure. From the plots it is evident that at finite *k*, the spin-wave frequencies propagating along two opposite directions differ significantly and this asymmetry increases with increase in the value of *k*. The results shown in Fig. 4(a,b) are modeled by using a modified dispersion relation for DE mode given by Eq. 2^19^,^20^,^21^, which takes into account of the shift in the frequency arising due to IDMI.


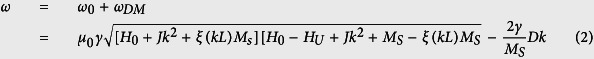


Here, g, *H*, *μ*_*0*_ and *M*_*s*_ carries the same meaning as used before in Eq. 1, *J* = 2*A*/*μ*_*0*_*M*_*s*_ the SW stiffness, *A* the exchange constant, *D* the IDMI constant, *H*_*U*_ = 2*K*_*U*_/*μ*_*0*_*M*_*s*_ the uniaxial anisotropy field (*K*_*U*_ the uniaxial anisotropy) and *ξ (kL*) = 1 − (1 − exp | − *kL*|)/|*kL*|, where *L* is the thickness of ferromagnetic film. The theoretical fit using Eq. 2 is shown by solid curves in Fig. 4(a,b). For *t* = 0.85 nm (Fig. 4(a)), using g = 2.0 from Kittel fit (as shown in Fig. 2) and *M*_*s*_ = 1030 kA/m from VSM (Kittel fit in Fig. 2 also yield similar value), the fitted experimental data yields *A* = 20 ± 2 pJ/m, *K*_*u*_ = (1.02 ± 0.05) × 10^5^ J/m^3^ and *D* = 0.25 ± 0.02 mJ/m^2^. The fitted value of *A* nearly agrees with the literature value. Following similar modeling, for *t* = 1.0 nm (Fig. 4(b)), these parameters are *g* = 2.0, *M*_*s*_ = 1100 kA/m, *K*_*u*_ = 1.45 × 10^5^ J/m^3^ and *D* = 0.21 ± 0.02 mJ/m^2^. As a matter of comparison we have added the symmetric dispersion as dashed curves in Fig. 4(a,b). These were generated using the standard dispersion relation for DE mode without considering IDMI i.e., by setting *ω*_*DM*_ = 0 in Eq. (2).

The interfacial DMI energy density can also be determined from the frequency difference (*Δf*) between spin-waves with opposite (+*k* and −*k*) wave-vectors as given by Eq. 3^19^,^20^,^21^.


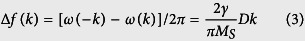


In this case the estimation of *D* is primarily determined by the experimentally measured quantities Δ*f, k* and *M*_*s*_. Figure 4(c,d) show the variation of Δ*f* with *k*, as obtained from measurements from samples with *t* = 0.85 nm and 1.0 nm, respectively. The slope of the linear fit to the data points for the sample with *t* = 0.85 nm gives *D* = 0.25 mJ/m^2^, whereas for the sample with *t* = 1.0 nm we obtain *D* = 0.21 mJ/m^2^ from the fit. Thus, the value of *D* is found to be consistent from two types of modeling of the experimental data. The observed increase in the *D* with the decrease in CoFeB thickness indicates the signature of an interfacial DMI at the W/CoFeB interface.

### Ferromagnetic layer thickness dependence of DMI constant

**Figure 5:**
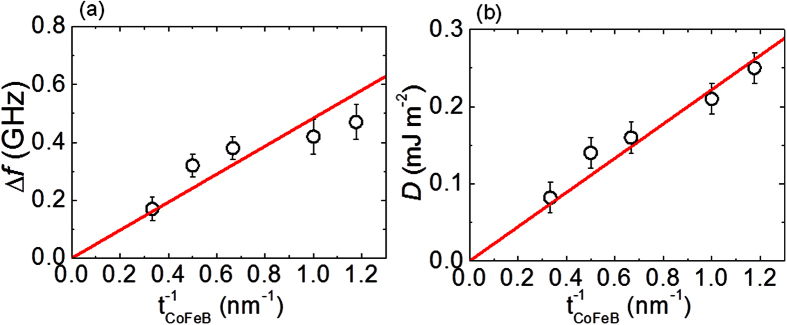
(**a**) Variation of asymmetry in the frequency and (**b**) Variation of DMI constant with the inverse of CoFeB thickness. The error bar in ∆*f* is shown by considering the error from the fitting of spectra as well as the instrumental resolution to determine the peak frequency and for *D* it is shown by taking into account the error in estimation of *M*_*s*_ as well as ∆*f*. The linear fits to the data in both the cases are shown using red solid lines.

In order to confirm that the observed asymmetry in the SW dispersion is induced purely by an IDMI at the W/CoFeB interface and not due to interface anisotropy at the asymmetric interface, we investigate the SW dispersion of samples with higher thicknesses of CoFeB. For this purpose, we discuss below the dependence of *Δf* and *D* on the thickness of the CoFeB layer. Displayed in Fig. 5(a,b) are the variation of *Δf* and *D* with the inverse of CoFeB thickness.

It is important to note from these plots that both *Δf* and *D* increases almost linearly with the decrease in the thickness of CoFeB and the nature of increase for both *Δf* and *D* are more or less the same. Linear fit is used to fit both CoFeB thickness dependence of *Δf* and *D*^30^. The apparent linear scaling of *D* with inverse of CoFeB thickness indicates that the asymmetry in the spin-wave dispersion originates primarily from the interfacial DMI. The asymmetry in *Δf* engendered due to interface anisotropy is known to scale as inverse square of ferromagnetic layer thickness^32^. We thus exclude the possibility of any contribution from interface anisotropy on the observed *Δf*. A remarkable feature to notice here is that neither *Δf* nor *D* start to deviate from the linear behavior with 1/*t* down to *t* = 0.85 nm, thus ensuring that the W/CoFeB interface does not degrade down to such small thickness values. In earlier studies, a sudden decrease was observed in *Δf* and *D* below ~1.6 nm of CoFeB thickness in Pt/CoFeB and this was explained by considering its origin in degradation of interface^23^. Non-degradation of W/CoFeB interface down to such small thickness is potentially very useful for the applications that aim to use DMI arising purely from interfacial origin such as fast domain wall velocity based spintronics devices.

### Asymmetry in magnon linewidth due to IDMI

Another manifestation of the presence of the interfacial DMI in the HM/FM/oxide heterostructures is an asymmetry in the dispersion of magnon lifetime with wave-vector *k*^33^. In order to get an insight into this asymmetry, we studied the variation in the linewidth of the spin-wave spectra with *k*. For that purpose, we performed a deconvolution of the linewdith for removing the contribution of the instrumental linewidth^34^.

**Figure 6:**
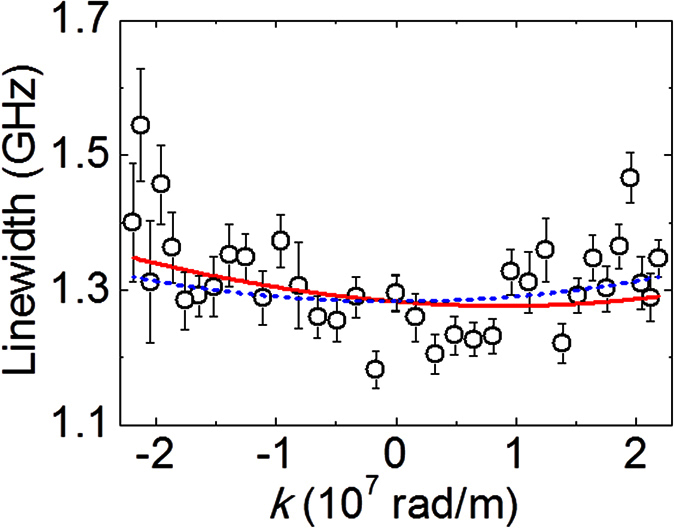
Linewidth of the spinwave spectra as a function of wave-vector for 2 W/1 CoFeB/2 SiO_2_ at a bias field of 0.1 T/μ_0_. Experimental linewidth data are denoted by symbols. Error bars show standard deviation of the spectral linewidth obtained using Lorentzian function. The solid red line represents the fitted curve using equation (4). Blue dashed line is the same without considering the DMI in the film stack.

Figure 6 displays the variation of linewidth i.e., in magnon lifetime with *k* at an applied magnetic field 0.1 T/μ_0_ for Sub/2 W/1 CoFeB/2 SiO_2_. Linewidth for spin-wave propagating in +*k* direction is smaller than the same for spin-wave propagating in −*k* direction, which indicates longer lifetime of magnon propagating in +*k* direction (c.f. Fig. 1(a)). The best fit to experimental variation of linewidth with wave-vector (as shown by continuous line in Fig. 6 is obtained using Eq. (4)^19^.


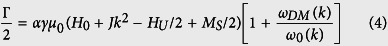


where Γ is the linewidth of the spin-wave spectra and other symbols denote same quantities as described earlier. We use *g* = 2.0, *M*_*s*_ = 1100 kA/m, *K*_*u*_ = 1.45 × 10^5^ J/m^3^ and *D* = 0.21 mJ/m^2^ and keep the damping coefficient α as fitting parameter. The best fit to the experimental data is obtained for α = 0.033 ± 0.002. Thus, Eq. 4 describes qualitatively the asymmetry in the linewidth due to the presence of DMI at the W/CoFeB interface. Figure 6 signifies that by using the same parameters as for fitting the dispersion curves in Fig. 4 we obtain a reasonably good fit for the linewidth of the spinwave spectra (magnon lifetime). Despite of the presence of significant error bar as accounted from the fit (due to relatively small IDMI value in comparison to the Pt based heterostructures), the observed asymmetry in the dispersion of magnon lifetime with wave-vector (c.f. Fig. 6) reconfirms the presence of IDMI primarily originating from W/CoFeB interface in the W/CoFeB/SiO_2_ film stacks.

## Discussion

In summary, we have investigated both qualitatively and quantitatively the pure interfacial DMI in W/CoFeB/SiO_2_ using BLS technique. The detailed study of magnetostatic surface spin-wave properties shows that the interfacial DMI leads to nonreciprocal spin-wave propagation, i.e., different properties for spin-waves propagating in opposite directions. In Damon-Eshbach geometry, by interchanging the magnetic field direction we observe asymmetry in the peak frequency, peak intensity and magnon lifetime. Furthermore, we showed that the IDMI constant scales as the inverse of CoFeB thickness down to 0.85 nm, indicating its origin as purely interfacial. The W/CoFeB interface shows almost negligible degradation down to sub-nanometer CoFeB thickness, which is highly desirable for technological applications those aim to use interface effect, in particular IDMI. Our investigation will be important for understanding the spin-wave dynamics as well as stabilizing DMI assisted skyrmion, soliton like domain wall motion, in CoFeB with W underlayer, which is known to have a large spin Hall angle.

## Methods

The thin film heterostructure Sub/*d* W/1 Co_20_Fe_60_B_20_/2 SiO_2_ with *d* = 1, 2, 3, 4, 5 nm and Sub/2 W/*t* Co_20_Fe_60_B_20_/2 SiO_2_ with *t* = 0.85, 1.0, 1.5, 2.0 and 3.0 nm (digits indicate thickness in nm) were deposited by dc/rf magnetron sputtering in Si (100) wafers coated with 100 nm SiO_2_. Purpose of varying W underlayer thickness was to select optimum W thickness, which results in significantly reduced magnetic inhomogeneity^35^,^36^ in the film stack. The base pressure of the deposition chamber was better than 2 × 10^−7^ Torr. CoFeB and W were grown using dc power of 28 Watt whereas the SiO_2_ was grown using rf power of 60 Watt at 13.56 MHz. All thin films were grown in Ar gas atmosphere of 1 mTorr pressure. Sputtering deposition condition (in particular the Ar deposition pressure and power) was optimized carefully to obtain the thin films in few nanometer and sub nanometer regime. A film stack Sub/1 Co_20_Fe_60_B_20_/2 SiO_2_ without W layer adjacent to CoFeB was used for reference measurement.

BLS study is performed using a Sandercock-type six pass tandem Fabry Perot interferometer^37^. Conventional back scattered geometry is used along with the provision of wave-vector selectivity to investigate the spin-wave dispersion relation. To obtain the wave-vector selectivity, the sample is rotated in vertical plane thus allowing access to various angles of incidence of laser on the sample. Details of the BLS set up can be found elsewhere^36^,^38^. For the larger incidence angles, the spectra were obtained after counting photons for several hours. It ensured that well defined spectra are obtained where the peak position can be estimated with accuracy of ~0.1 GHz. We use free spectral range of (FSR) 50 GHz and a 2^9^ multi-channel analyser. The frequency resolution is determined by estimating FSR/2^9^ (≈0.1 GHz) for the Stokes and anti-Stokes peaks of the BLS spectra^21^,^23^. The instrumental linewidth of our setup is ~0.3 GHz as obtained from the analysis of the elastic peak^34^ obtained in our experiment.

## Additional Information

**How to cite this article**: Chaurasiya, A. K. *et al.* Direct Observation of Interfacial Dzyaloshinskii-Moriya Interaction from Asymmetric Spin-wave Propagation in W/CoFeB/SiO2 Heterostructures Down to Sub-nanometer CoFeB Thickness. *Sci. Rep.* **6**, 32592; doi: 10.1038/srep32592 (2016).
